# Supplementary material for: Disseminating implementation science: Describing the impact of animations shared via social media
Source: PLoS One. 2022 Jul 7;17(7):e0270605. doi: 10.1371/journal.pone.0270605 (PMC9262190; doi:10.1371/journal.pone.0270605)
Supplement: S1 File — (DOCX) [file pone.0270605.s002.docx]

**S1 File: The brief developed by MS, LC and JC**

**Enhancing audit and feedback**

Stylistically, the animation is to be engaging, accessible and presented with a positive, improvement-focussed tone.

**Common content in both animations:**

Some patients do not receive ‘best care’. To improve care, hospitals undertake ‘audit and feedback’.

Audit and feedback involves giving people a summary of their performance over a specified period of time. It is used a lot in healthcare to improve the quality of care.

Audit and feedback has different components, like assessing practice, providing feedback, making changes. These components can be done in different ways.

How these components are done can affect whether, and by how much, audit and feedback improves care.

To find ways to increase improvement from audit and feedback, we looked at how hospitals are currently doing audit and feedback. We studied six hospitals within four diverse NHS organisations We used interviews, observations and documentary analysis, and repeatedly presented emergent findings to stakeholders.

**National audit:**

The National Audit of dementia seeks to improve the care for people with dementia.

Almost all (98%) of hospitals take part in the National Audit of Dementia.

More than one in four people in acute hospitals have dementia but they don't always get the needed care.

We explored how the national audit of dementia is undertaken to find out how to enhance its impact on the improvement of patient care.

We studied how six hospitals undertake the national audit.

We used interviews, observations and documentary analysis, and repeatedly presented emergent findings to stakeholders.

We found that the national report was sent back to the clinical leads who then turn the national report into an internal report which it was given to Trust Level committees. The internal report described the audit and included a draft action plan.

Issues

We found that the feedback was slow to arrive and did not reach people providing care, instead the feedback stayed at an organisational level.

Hospital dementia leads described the national report as being confusing. They also said they were unsure how to lead hospital-wide improvement.

Hospital dementia leads used the audit report to produce an action plan for the organisation. [Setting: Office]

The action plans focused on the care in national recommendations (e.g. improve nutritional care), often disregarding low local performance in other aspects of care (e.g. assessing risk of pressure sores).

We found that there was little exploration of why performance was low.

The clinical leads suggested actions based on what they could do (like training or more audit), rather than actions that addressed the causes of performance.

We think the hospital dementia leads need support and time to implement changes from the national audit report.

We have developed a way to support the clinical leads to improve care. We called this, ‘Logical Improvement Planning’.

Logical Improvement Planning aims to help the clinical leads analyse hospital performance and consider the impact on hospital priorities.

This involves helping them to select actions based upon an analysis of the hospital data and the causes of performance. [Setting: Ward] It also involves describing what we found to influence organisational commitment to implement the changes. [Setting: Board room]

**CALL TO ACTION:** For more information about Logical Improvement Planning, see links on Twitter @StudyImprove or contact michael.sykes@ncl.ac.uk

**Ward audit:**

Ward audit is a form of audit and feedback which involves ward managers regularly receiving feedback about the clinical performance of staff on their ward.

Ward audit feedback includes information about the performance of clinicians at things like:

- medicine management (e.g. recording the giving of drugs),
- infection control (e.g. checking if the ward has been cleaned),
- nutrition (e.g. whether patient needs have been assessed),
- bladder and bowel care (e.g. writing when a patient’s catheter was inserted)
- communication (e.g. whether discharge has been discussed with carers or relatives). [Setting: Patient bedside]

We explored how ward audit is undertaken to find out how to enhance its impact on the improvement of patient care.

We found that at three sites clinical staff extracted data from the clinical record, and sometimes did interviews with patients or staff and observed how care was given. Ward managers knew the results when they were entered, and got analysed data within a week. The analysed data was colour coded, typically red, amber and green.

Issues

At one of the hospitals, it was noticed that manually gathering the data took 5 hours per ward per month. This was done by band 5 to 7 nurses. Staff prioritised data collection rather than taking care of the patients. [Setting: On ward e.g. Looking in patient notes / speaking / observing staff and patients]

The data that was collected was not always accurate, a reason for this was the fear of feedback.

The feedback that got back to the staff was often received as punitive. [Setting: Office]

- “it would be like the house of cards that was toppled from the top and it would be all down. So, [matron] would be under pressure and she would put that pressure on me” (Ward manager)
- “We were told it…was a massive failure on our part as a ward.” (staff nurse)
- Staff at different levels did not think that the data was accurate. This meant that the data was not used to develop improvement actions.
- To improve the data hospitals:
- Audited the same topic in a different way, for example, by the infection control nurse.
- Bought electronic tablets to record the data more quickly
- Introduced ‘ward accreditation’, where a member of staff who was not from the ward collected data. [Setting: Office]

A requirement to undertake ‘low value’ work may undermine staff attitudes towards the quality of care. Punitive feedback may decrease staff morale, increase staff turnover and reduce the quality of care. [Setting: Ward]

Solutions

We propose an intervention to:

- discuss the accuracy of ward monitoring data and the extent it leads to changes likely to improve care,
- explore local perceptions of the feedback within ward monitoring audit and other forms of clinical performance management,
- explore causes of punitive feedback, [Setting: Office]
- consider implications from punitive feedback (e.g. staff morale, collective leadership, ability to find high-performing wards that might have lessons to share, ability to develop hospital-level interventions) [Setting: Committee table]

**CALL TO ACTION:** There is an opportunity to improve care by changing the way ward monitoring feedback is given. To get involved in a study to enhance ward audit, see links on Twitter @StudyImprove or contact michael.sykes@ncl.ac.uk

**AT THE END OF EACH:**

Research funded by the National Institute for Health Research (NIHR) (Michael Sykes’ Doctoral Fellowship 2016-09-028)

This publication presents independent research funded by the National Institute for Health Research (NIHR). The views expressed are those of the author and not necessarily those of the NHS, the NIHR or the Department of Health and Social Care.

[LOGO ATTACHED]
