# Supplementary material for: Disseminating implementation science: Describing the impact of animations shared via social media
Source: PLoS One. 2022 Jul 7;17(7):e0270605. doi: 10.1371/journal.pone.0270605 (PMC9262190; doi:10.1371/journal.pone.0270605)
Supplement: S1 Fig — (DOCX) [file pone.0270605.s003.docx]

**S1 Fig:** **The unadjusted statistical process control c-chart showing weekly accesses of Sykes et al (2021)**

2 s.d.

3 s.d.
