# Supplementary material for: Disseminating implementation science: Describing the impact of animations shared via social media
Source: PLoS One. 2022 Jul 7;17(7):e0270605. doi: 10.1371/journal.pone.0270605 (PMC9262190; doi:10.1371/journal.pone.0270605)
Supplement: S1 Table — (DOCX) [file pone.0270605.s004.docx]

**S1 Table: Weekly data per article**

|  | **ARTICLE 1** | **ARTICLE 2** | |
| --- | --- | --- | --- |
| **Week** | **Weekly increase** | | **Views** |
| 1 | 33 | 39 | n/a |
| 2 | 26 | 72 | n/a |
| 3 | 25 | 45 | n/a |
| 4 | 17 | 46 | n/a |
| 5 | 27 | 32 | n/a |
| 6 | 31 | 29 | n/a |
| 7 | 20 | 39 | n/a |
| 8 | 4 | 28 | n/a |
| 9 | 10 | 31 | n/a |
| 10 | 26 | 36 | n/a |
| 11 | 21 | 43 | n/a |
| 12 | 29 | 42 | n/a |
| 13* | 21 | 66 | 871 |
| 14 | 28 | 11 (69**) | 1749 |
| 15 | 25 | 99 (41**) | 1805 |
| 16 | 16 | 23 | 1824 |
| 17 | 14 | 36 | 1838 |
| 18 | 14 | 82 | 2234 |
| 19 | 20 | 33 | 2263 |
| 20*** | 67 | 29 | 2279 |
| 21 | 43 | 59 | 2288 |
| 22 | 25 | 32 | 2293 |
| 23 | 21 | 59 | 2306 |

* Article 2 animation released before this week

** Adjusted data

*** Article 1 animation released before this week

n/a = Not applicable as the animation had not been released
