# Supplementary material for: Disseminating implementation science: Describing the impact of animations shared via social media
Source: PLoS One. 2022 Jul 7;17(7):e0270605. doi: 10.1371/journal.pone.0270605 (PMC9262190; doi:10.1371/journal.pone.0270605)
Supplement: Attachment — Submitted filename: PONE-D-22-05141_reviewed.pdf [file pone.0270605.s005.pdf]

PLOS ONE

Disseminating implementation science: Describing the impact of social-media delivered animations

--Manuscript Draft--

**Manuscript Number:**	PONE-D-22-05141
**Article Type:**	Research Article
**Full Title:**	Disseminating implementation science: Describing the impact of social-media delivered animations
**Short Title:**	Disseminating implementation science: Describing the impact of social-media delivered animations
**Corresponding Author:**	Michael Sykes<br>Northumbria University<br>Newcastle upon Tyne, United Kingdom (+44) UNITED KINGDOM
**Keywords:**	Dissemination; Animation; Social media; Statistical process control
**Abstract:**	Background<br><br>Dissemination is an under-researched activity that is important to researchers and funders and may have a role in creating conditions for implementation. We aim to study the impact of two social-media delivered animations upon dissemination.<br><br>Methods<br><br>We developed two short animations to increase the awareness of healthcare positional leaders of work undertaken to investigate a much-used implementation intervention. We measured both how frequently the related articles were accessed and engagement with the research team, before and after the intervention. We analysed variation over time using statistical process control to identify both trend and periods of significant change.<br><br>Results<br><br>We found evidence that the animation increased how frequently the articles were accessed, with a significant increase (p = <0.01) during the two weeks after release of each animation. One animation was associated with an increase in positional leaders' engagement with the research team.<br><br>Conclusions<br><br>We found that social media delivered animations can enhance dissemination. We describe lessons from the work to develop the intervention and support calls for work to increase the understanding and adoption of effective dissemination interventions. Our findings provide support for further work using randomised study designs.
**Order of Authors:**	Michael Sykes
	Lucia Cerda
	Juan Cerda
	Tracy Finch
**Additional Information:**	
**Question**	**Response**
**Financial Disclosure**<br><br>Enter a financial disclosure statement that describes the sources of funding for the	This report is independent research arising from a Doctoral Research Fellowship (DRF-2016-09-028) supported by the National Institute for Health Research (NIHR). The views expressed in this presentation are those of the authors and not necessarily those of the NHS, the National Institute for Health Research or the Department of

Powered by Editorial Manager® and ProduXion Manager® from Aries Systems Corporation

work included in this submission. Review the submission guidelines for detailed requirements. View published research articles from *PLOS ONE* for specific examples.<br><br>This statement is required for submission and **will appear in the published article** if the submission is accepted. Please make sure it is accurate.<br><br>**Unfunded studies**<br>Enter: *The author(s) received no specific funding for this work.*<br><br>**Funded studies**<br>Enter a statement with the following details:<br>• Initials of the authors who received each award<br>• Grant numbers awarded to each author<br>• The full name of each funder<br>• URL of each funder website<br>• Did the sponsors or funders play any role in the study design, data collection and analysis, decision to publish, or preparation of the manuscript?<br>• **NO** - Include this sentence at the end of your statement: *The funders had no role in study design, data collection and analysis, decision to publish, or preparation of the manuscript.*<br>• **YES** - Specify the role(s) played.<br><br>* typeset	Health and Social Care.
**Competing Interests**<br><br>Use the instructions below to enter a competing interest statement for this submission. On behalf of all authors, disclose any competing interests that could be perceived to bias this work—acknowledging all financial support and any other relevant financial or non-financial competing interests.<br><br>This statement is required for submission and **will appear in the published article** if the submission is accepted. Please make sure it is accurate and that any funding sources listed in your Funding Information	The authors have declared that no competing interests exist.

later in the submission form are also declared in your Financial Disclosure statement.<br><br>View published research articles from *PLOS ONE* for specific examples.<br><br>**NO authors have competing interests**<br><br>Enter: *The authors have declared that no competing interests exist.*<br><br>**Authors with competing interests**<br><br>Enter competing interest details beginning with this statement:<br><br>*I have read the journal's policy and the authors of this manuscript have the following competing interests: [insert competing interests here]*<br><br>* typeset	
**Ethics Statement**<br><br>Enter an ethics statement for this submission. This statement is required if the study involved:<br><br>• Human participants<br>• Human specimens or tissue<br>• Vertebrate animals or cephalopods<br>• Vertebrate embryos or tissues<br>• Field research<br><br>Write "N/A" if the submission does not require an ethics statement.<br><br>General guidance is provided below. Consult the submission guidelines for detailed instructions. **Make sure that all information entered here is included in the Methods section of the manuscript.**	The study was approved by the Newcastle University Ethics Committee (Ref: 3917/2020). Informed consent was not sought as we used publicly available, anonymised data.

**Format for specific study types**<br><br>**Human Subject Research (involving human participants and/or tissue)**<br>• Give the name of the institutional review board or ethics committee that approved the study<br>• Include the approval number and/or a statement indicating approval of this research<br>• Indicate the form of consent obtained (written/oral) or the reason that consent was not obtained (e.g. the data were analyzed anonymously)<br><br>**Animal Research (involving vertebrate animals, embryos or tissues)**<br>• Provide the name of the Institutional Animal Care and Use Committee (IACUC) or other relevant ethics board that reviewed the study protocol, and indicate whether they approved this research or granted a formal waiver of ethical approval<br>• Include an approval number if one was obtained<br>• If the study involved *non-human primates*, add *additional details* about animal welfare and steps taken to ameliorate suffering<br>• If anesthesia, euthanasia, or any kind of animal sacrifice is part of the study, include briefly which substances and/or methods were applied<br><br>**Field Research**<br><br>Include the following details if this study involves the collection of plant, animal, or other materials from a natural setting:<br>• Field permit number<br>• Name of the institution or relevant body that granted permission	
**Data Availability**<br><br>Authors are required to make all data underlying the findings described fully available, without restriction, and from the time of publication. PLOS allows rare exceptions to address legal and ethical concerns. See the PLOS Data Policy and FAQ for detailed information.	Yes - all data are fully available without restriction

A Data Availability Statement describing where the data can be found is required at submission. Your answers to this question constitute the Data Availability Statement and **will be published in the article**, if accepted.<br><br>**Important:** Stating 'data available on request from the author' is not sufficient. If your data are only available upon request, select 'No' for the first question and explain your exceptional situation in the text box.<br><br>Do the authors confirm that all data underlying the findings described in their manuscript are fully available without restriction?	
**Describe where the data may be found in full sentences. If you are copying our sample text, replace any instances of XXX with the appropriate details.**<br><br>• If the data are **held or will be held in a public repository**, include URLs, accession numbers or DOIs. If this information will only be available after acceptance, indicate this by ticking the box below. For example: *All XXX files are available from the XXX database (accession number(s) XXX, XXX.).*<br>• If the data are all contained **within the manuscript and/or Supporting Information files**, enter the following: *All relevant data are within the manuscript and its Supporting Information files.*<br>• If neither of these applies but you are able to provide **details of access elsewhere**, with or without limitations, please do so. For example:<br><br>*Data cannot be shared publicly because of [XXX]. Data are available from the XXX Institutional Data Access / Ethics Committee (contact via XXX) for researchers who meet the criteria for access to confidential data.*<br><br>*The data underlying the results presented in the study are available from (include the name of the third party*	All relevant data are within the manuscript and its Supporting Information files.

Powered by Editorial Manager® and ProduXion Manager® from Aries Systems Corporation

and contact information or URL).<br>• This text is appropriate if the data are owned by a third party and authors do not have permission to share the data.<br><br>* typeset	
Additional data availability information:	Tick here if the URLs/accession numbers/DOIs will be available only after acceptance of the manuscript for publication so that we can ensure their inclusion before publication.

Powered by Editorial Manager® and ProduXion Manager® from Aries Systems Corporation

Manuscript
Click here to access/download;Manuscript;Animation paper v1_Plos.docx

Disseminating implementation science: Describing the impact of social-media delivered animations

Michael Sykes [1]*, Lucia Cerda [2], Juan Cerda [2], Tracy Finch[1]

[1] Department of Nursing, Midwifery and Health, Northumbria University, Newcastle upon Tyne, United Kingdom

[2] Lucia Cerda Design, Barcelona, Spain

* Corresponding author

E-Mail: Michael.sykes@northumbria.ac.uk (MS)

Abstract

Background: Dissemination is an under-researched activity that is important to researchers and funders and may have a role in creating conditions for implementation. We aim to study the impact of two social-media delivered animations upon dissemination.

Methods: We developed two short animations to increase the awareness of healthcare positional leaders of work undertaken to investigate a much-used implementation intervention. We measured both how frequently the related articles were accessed and engagement with the research team, before and after the intervention. We analysed variation over time using statistical process control to identify both trend and periods of significant change.

Results: We found evidence that the animation increased how frequently the articles were accessed, with a significant increase ($p = <0.01$) during the two weeks after release of each animation. One animation was associated with an increase in positional leaders' engagement with the research team.

Conclusions: We found that social media delivered animations can enhance dissemination. We describe lessons from the work to develop the intervention and support calls for work to increase the understanding and adoption of effective dissemination interventions. Our findings provide support for further work using randomised study designs.

Key words: Dissemination; Animation; Social media; Statistical process control.

Introduction

Funders expect publicly-funded researchers to undertake activities to disseminate their research to multiple research users as part of the work to develop impact ~~(e.g.~~ [1; 2; 3]~~). The f~~unders often provide resource to support dissemination of findings, and many provide structures to transmit the findings ~~(e.g.~~ [4; 5]~~)~~.

Wilson et al [6] found that dissemination was rated as important or very important by 93% (n=216) of respondents to their survey of principal investigators of applied and public health research. The most common reported communication channel was through academic journals (98%) and conference presentations (96%). The use of animation or social media was not included in the list of options in their 2003-8 study, although 5% referred to using 'other' communication channels and one respondent described the use of a DVD.

Capturing the impact of dissemination activities is important to funders (e.g. [7]) and provides the opportunity for researchers to monitor and improve effectiveness of dissemination activities. The number of times a paper has been accessed can be used as a measure of 'user pull' uptake of research [8], and provides a measure of dissemination [9; 10].

Dissemination is, "a planned process that involves consideration of target audiences and the settings in which research findings are to be received" (p2; [6]). Nilsen [11] describe a diffusion-dissemination-implementation continuum, where "dissemination is the active spread of new practices to the target audience using planned strategies"(p2). Dissemination activity may address antecedents of behaviour change, including awareness, knowledge, perceptions, and motivation [12]. Baker et al [12] called for research that extends dissemination science and practice.

The effect of dissemination interventions might be influenced by components to the intervention, the skills of those developing the intervention, influences upon the medium of delivery and the wider context; as such, dissemination interventions are complex interventions (e.g 13). Guidance recommends that those developing complex interventions consider the research perspective, incorporate evidence, theory and stakeholder views; consider implementation and use iterative design methods (e.g. 13; 14).

McGuire's Persuasive Communication Matrix [15] proposed five dissemination variables: the channel, source, message, audience, and setting. Social media is a valuable channel for dissemination and is associated with increased downloads and citations [16; 17]. Social media enables the use of more accessible content (for example, through using more visual presentation style; [18] and can provide information through a route that is more consistent with how clinical staff access information (for example, on a smartphone rather than through articles viewed on a hospital computer [19]). Twitter is the most commonly studied social media platform in correlational studies seeking to increase the impact of health research [20] and a valuable social media channel for health research [21]. Studies of the effect of Tweets alone (without animation) have found that they did not increase downloads or citations [22; 23]; Adding infographics or podcasts to promotional tweets may increase article accesses [24].

The current paper describes work to study the impact of two social-media delivered animations upon dissemination. The intervention targets positional leaders in healthcare through the use of a Twitter-delivered animation. The source was an applied health researcher, describing work undertaken as part of a PhD to describe and enhance audit and feedback. The work was undertaken with a supervisory team, and involved substantial stakeholder involvement through co-production and advisory groups [25; 26]. The message of the first animation was a multisite description of what currently happens when a national audit reaches the hospital and how it could be enhanced through

an intervention to support recipients to analyse performance, select strategies and generate commitment. The message of the second animation was a multisite description of what happens during an audit of the quality of care on wards across a hospital. Both messages were delivered through the medium of animation, a form of "non-text output" [27] and time-based visual artwork that illustrates a story unfolding over time [28]. The target audience for both animations was positional healthcare leaders (for example, directors, clinical audit leads). It was anticipated that they would be viewing social media in work, home or travel settings. In this paper we describe the effect of the animation upon the frequency with which particular open-access papers [25; 26] were accessed.

Materials and methods

Aim

To study the impact of two social-media delivered animations upon dissemination.

Study design and theoretical framework

This is a phase II [29] before-and-after study using statistical process control to describe the impact of an animation intervention upon the weekly number of accesses of the target articles (Appendix B presents the CONSORT checklist). We conceptualise dissemination as a process, an outcome of which is whether an article subsequently accessed. We propose that whether the article is cited or acted upon is influenced by the article content, rather than the dissemination activity. We recognise that the accessing of articles will vary over time (e.g. due to holiday periods); by monitoring the significance of variation we are able to describe the impact of the animation as an event in the dissemination process [30].

Setting and participants

This was an online study where participants were social media (Twitter) users. The source Twitter account had approximately 530 followers, with further participants able to view the tweet as a result of likes, retweets and searches. The followers were predominantly described in their biographies as healthcare improvement leaders, clinicians, clinical positional leaders, clinical academics and people interested in implementation science.

Intervention

The animations, which are described in Box 1 and in the TIDieR checklist (Appendix C), are available at: https://twitter.com/Msykes09/status/1330556748210515968?s=20 and https://twitter.com/Msykes09/status/1408675290176491521?s=20.

Intervention development involved initial scoping of the content to be delivered, consideration of the message, the audience, the channel and the style, followed by iterative stakeholder engagement and refinement: MS summarised the target articles to JC and LC who asked exploratory questions in relation to the audience and key messages for audience sub-groups. These questions illuminated both that the target audience was chosen as they were anticipated to have influence over the proposed enhancements, and that the animations needed to be acceptable, accessible and engaging. MS proposed delivery via Twitter as the primary mode of delivering the animations. MS highlighted the role of 'tone' in developing an acceptable animation, notably that the animation should be descriptive, non-critical and offer solutions. Questions from JC and LC explored the language being used, towards the goal of making the message accessible to the audience. MS, JC and LC also explored how to make the animation engaging, resulting in the illustration of scenes familiar to the target audience (e.g. bedside, office, committee room), the careful use of movement, paying attention to the cognitive load and describing the source of the information, which was anticipated to be credible. Following initial discussion between MS, JC and LC, MS produced a brief for the content of the two animation (Appendix A). Based upon the brief, JC and LC produced a storyboard for each presentation, upon which MS gave comments. The amended storyboards were then shared by email with the co-authors of the target papers who provided further group feedback during a face-to-face meeting. This led to changes to simplify the text and increase diversity in the images. The storyboards were amended again and shared with the co-production group (three clinical leads, three hospital clinical audit leads and three carers) during a virtual meeting. This led to changes in language (e.g. changing 'sites' to 'hospitals'). The amended storyboards were shared by email to further stakeholders, including implementation scientists (n=8) and national audit leads (n=4). The storyboards were refined again, so as to add the learning outcomes. The final version was shared with the funder in line with contractual requirements. JC and LC produced two animations based upon the revised storyboards. The draft animations were shared with the supervisory team and the co-production group and refined based upon their feedback. This led to changes both to the length of each scene and when text was added to each scene.

**The source:** An applied health researcher, describing work undertaken as part of a PhD to describe and enhance audit and feedback in dementia care. The work was undertaken with a supervisory team, and involved substantial stakeholder involvement through co-production and advisory groups [25; 26].

The channel: Twitter account with approximately 530 followers

Article 1 message: A description of what currently happens when a national audit reaches the hospital and how it could be enhanced through an intervention to support recipients to analyse performance and select strategies.

Article 2 message: A description of what currently happens during a nurse-led hospital-wide audit of the quality of care in wards and steps to enhance the audit.

The audience: Positional healthcare leaders (for example, directors, clinical leads). For article 1, they were targeted through a Tweet directed to regional and national organisations leading healthcare improvement (e.g. @TheIHI @FabNHSStuff @ImprovementCym @Aqua_NHS @Improve_Academy), national improvement and regulation agencies (@HIQA @HSCQI @noca_irl @online_his) and an umbrella organisation for English hospital providers (@NHSConfed). For article 2, they were targeted through Tweets directed to national nursing leads (e.g. @CNOEngland, @CharlotteMcArdl), directors of nursing and nursing research leaders (e.g. @AlisonProf, @DrJoanne_Cooper)

The setting: It was anticipated that they would be viewing social media on a phone or tablet in work, home or travel settings.

Box 1: A description of the animation content and delivery

The animations were developed to be engaging: They included content to support the viewer to compare the findings to their own practice, by describing current practice and asking whether this "sounds like what happens at your hospital"; They included content relating the work to priorities, by presenting benefits for patients, clinicians and positional leaders. The animations used accessible language and employed a positive, improvement-focussed tone intended to be acceptable to recipients. It was anticipated that the describing the funder, university and stakeholder involvement would develop perceived credibility and trustworthiness.

The animations were released at different times of the year. Animation 1 was tweeted three times, with the link to the article in a linked tweet and the first author's biography.

Animation 1 was tweeted by MS on 17th November 2020: *Here's a short animation of our @NIHRresearch funded work to describe and enhance a national audit. The work was undertaken with @TracyLFinch @niinamk @drlouiseallan and Richard Thomson #NUPHSI. It would not have been possible without the input from a large number of stakeholders, including @afMetaLab and many others whom I am unable to name here.*

Animation 1 was tweeted again by MS on 22nd November 2020: *Enhancing what happens when a national audit reaches the hospital. May be of interest to @THIS_Institute @TheIHI @FabNHSStuff @HIQA @HSCQI @noca_irl @online_his @ImprovementCym @Aqua_NHS @Improve_Academy @FNightingaleF @NHSConfed*

Animation 1 was tweeted again by MS on 23rd November 2020 with *#CAAW20*, in order to bring it to the attention of people following the Clinical Audit Awareness Week: *Those following #CAAW20 may be interested in this.*

Animation 2 was tweeted by MS on tweeted on 26th June 2021: *NEW animation: Describing a monthly audit of ward quality at NHS hospitals.* The thread continued: *You can read more about what we did, what we found and what we propose here:* [Link to paper]

Animation 2 was tweeted by MS on 2nd July 2021: *Directors of nursing, matrons + ward managers described ward audit data as 'meaningless' & 'a sea of green'. @DavidFMelia @RivkahMiar @Antonialynch @mapFlynn @AlisonSmith2306 The animation & paper describe that the audit is costly, may have adverse effects & could be enhanced @Karen_Goudie @Day2H @lzredfernsoecno @KarenDunderdale @LeesLizzie @angelawooduk @PeteWRN*

Animation 2 was tweeted by MS on 4th July 2021: *The paper and animation describe the discomforting issue of punitive feedback and its impacts upon patient care, staff wellbeing, improvement, assurance and cost.*

Animation 2 was tweeted by MS on 13th July 2021: *A multi-site study using interviews, observations & doc analysis, supported by 2 groups of stakeholders. Funded by @NIHRresearch Found opportunities to improve care, staff well-being, costs & assurance. @AlisonProf @DrJoanne_Cooper @jorycroftmalone @PorteousDr @Evidence4QI*

Data collection

The primary and target behavioural outcome of the animation was the number of times the paper had been accessed. This was retrieved from the target journals' website. We captured events that may confound the results (e.g. conferences, newsletter or non-study tweets). The secondary outcome was direct requests to the first author for further information.

Analysis

Statistical process control (SPC) charts describe system performance over time. We used SPC charts to describe the weekly number of accesses of the target papers before and after the animation. In line with guidance [30], we labelled the SPC with events in the dissemination process (potential confounders), for example, when the work described in article 1 was presented by the research

team at an international conference, or when article 2 was highlighted in a newsletter independent of the research team.

The weekly number of accesses was analysed using an Excel-based statistical process control c-chart tool [30]:

$$\bar{c} = \frac{\sum_{i=1}^{m} x_i}{m}$$

$$Control\ Limits = \bar{c} \pm 3\sqrt{\bar{c}}$$

The trend in the first three weeks after publication demonstrated special cause variation and, in line with guidance for the use of SPC, was removed from the graph [31]. The SPC charts describe the subsequent trend for the period before and after the release of the animations. Visual analysis of the SPC charts sought temporal association between the intervention and the primary outcome (article accesses) and secondary outcome (contacts to corresponding author).

Results

Statistical process control charts are interpreted by looking for occasions when frequency line crosses the standard deviation line. Crossing the three standard deviation line is evidence for significant difference (p=<0.01), and is referred to as special cause variation. Remaining within the standard deviation line is evidence that whilst there might be variation, this is not significantly different from prior frequency and is referred to as common cause variation.

The mean number of weekly accesses for article 1 was 25, with a significant decrease evident during the last week of August. In the two weeks after the intervention, there was a significant increase in the weekly number of accesses, as described in figure 1. There was common cause variation (that is, no significant change) after presenting the paper at an international conference or by tweeting links to the paper without the animation during weeks 2 and 13.

The mean number of weekly accesses for article 2 was 43, with a significant decrease evident during the third week of July. In the two weeks after the intervention, there was a significant increase in the weekly number of accesses, as described in figure 2. Article 2 also had significant increases in the number of accesses following publicity about the paper by @Evidence4QI (a project seeking to implement evidence in quality improvement projects) and after posting in the Q Community (https://q.health.org.uk/) discussion forum. Whilst study tweets were re-tweeted; we sought but did not identify novel non-study tweets.

Fig 1. A statistical process control c-chart showing weekly accesses of Sykes et al (2020) and numbers of contacts [c] made to first author by stakeholders

Fig 2. A statistical process control c-chart showing weekly accesses of Sykes et al (2021) and numbers of contacts [c] made to first author by stakeholders

The secondary outcome was direct requests to the first author for further information. We received seven requests for further information about article 1 during the study period. Five of the requests came during the 8 days after the animation was released.

27th July

Request to speak to a deputy director of nursing about the study

13th October

Request to speak to a national clinical lead about the work

19th November

Invite to speak to a Trust-level committee

21st November

Invite to speak to a national network of clinical audit leads

Contact from the clinical lead of a national audit

24th November

Invite to speak to a Trust-level committee

25th November

Request to use the animation in a presentation by a non-executive to a Trust-level committee

We received five requests for further information about article 2 during the study period, one of which came in the period immediately after the release of the animation:

26h June

Request to speak to an improvement lead from a UK healthcare provider organisation

Discussion

There is evidence that the animation intervention initiated the intended response in the target audience. We found that each animation was associated with a significant increase in the weekly number of accesses. Specifically, during the two weeks after the intervention there was a significant

increase. Over these two weeks, there were 60 accesses above the mean for the proceeding period for article 1 and 24 accesses above the mean for the proceeding period for article 2 . There is evidence that the animation 1 was temporally associated with new requests for information and discussion from the target audience.

There are strengths and limitations to the work: The study describes a feasible dissemination intervention developed by an early career researcher. Consistent with McGuire's Persuasive Communication Matrix, we identified the source, channel, message, audience and setting [15]. The animations were designed to meet a specific outcome (for viewers to be motivated to gain more information about the research described in the animation) and to meet specific dissemination outcomes (to be engaging, accessible and acceptable). The iterative, multi-method stakeholder engagement is a further strength. The overall effect of the engagement was to support the use of animations delivered via Twitter, and both to refine delivery and focus the message so that messages perceived as being more peripheral were removed. This highlights the role of stakeholder engagement in identifying the messages to be disseminated.

It is possible that the findings are not generalisable to different animations, articles or audiences. Article 1 was published in an open-access journal (Implementation Science). Implementation Science is the third ranked health policy journal [32] with a 2-year impact factor of 4.525 [33]. Article 2 was published in an open-access journal (BMC Health Services Research), the 66th ranked health policy journal [32] with a 2-year impact factor of 2.655 [34]. It is possible that accesses would have been reduced if access were behind a paywall, or if published in a different journal. Data collection was extracted manually from the journal website; like previous studies (e.g. [10]) we are not able to confirm the accuracy of the count. During data collection for the second animation, the counter did not change during the period 2nd to 5th July (cumulative 871 accesses) and 6th to 13th July (cumulative 882 accesses). These were the only multi-day periods when the journal counter recorded that the articles were not accessed; this hiatus was followed by a large increase (88 accesses). It is anticipated that this was an error in the data collection by the journal. Email correspondence with the journal sought, unsuccessfully, to correct this. As a result, the data analysis included an estimate for data distribution for this two week period based upon article 1. For transparency, the uncorrected data is presented in Appendix 2 (Figure 3). Both the corrected and uncorrected graphs illustrate a significant increase in accesses in two of the three weeks after the release of the second article. Consistent with the guidance [31; 35], we re-calculated the mean and standard deviation after the initial period of special cause variation.

The current study focussed on usual dissemination and the online delivery of animations targeted at positional leader knowledge users. The main outcome was for recipients to seek further information,

as measured through article access. It cannot be inferred that those accessing the paper where the target audience or that they behaved differently as a result of accessing the article. However, subsequent contacts were from members of the target audience. The animations cost £508; we did not monitor time costs, but these were estimated to be 25 hours. The animation intervention was associated with a significant increase in accesses that lasted two weeks. Future work should consider how to enhance the response, for example, a multi-faceted approach [36] may result in a more sustained increase. Future research should also consider the link between increased article accessed and author contact, and impact upon target audience behaviour and patient outcomes.

There were important lessons in developing the animations. The mode of delivery impacted upon number and content of the scenes: there is a 140 second limit to embedded videos on Twitter and we found there was a minimum length of time needed to understand a scene. This constrained the number and cognitive load of scenes, including the volume of text and use of movement. In developing the animation, the role of movement was a key consideration: we sought to balance the use of movement to gain attention with the impact of movement on cognitive load that could distract from participant engagement with the message. The animation was anticipated to be viewed on tablets and mobile phones, which impacted upon the size of the font. It was anticipated that this could be viewed in work, public or home settings, as such sound needed to be optional. Providing both text and voice may have added to the cognitive load; As a result, we opted for gentle music consistent with the intended tone rather than narration. The feedback from the consultation with stakeholders highlighted opportunities to increase accessibility by improving the clarity about the research findings and addressing international differences in how audiences may view the findings.

Conclusion

We studied the impact of animations to increase the frequency with which two open-access papers [25; 26] were accessed. In designing the intervention, we drew upon McGuire's Persuasive Communication Matrix [15] to consider the source, channel, message, audience, and setting. We found that the release of the animation was associated with a significant increase in the number of times the article was accessed. We observed that neither tweeting article links without the animation nor presentation at an international conference led to a significant increase. We propose that the use of animation distributed via Twitter may provide an effective way to disseminate research findings and increase stakeholder engagement with study findings. Further work to test the use of animation using randomised study design, and to investigate the impact upon dissemination outcomes from evidence newsletters and delivering animations via professional digital forums, would be valuable.

Abbreviations

NHS – National Health Service

s.d. – Standard deviation

MS – Michael Sykes

LC – Lucia Cerda

JC – Juan Cerda

TF – Tracy Finch

Declarations

Ethics approval:

The study was approved by the Newcastle University Ethics Committee (Ref: 3917/2020)

Consent for publication

Not applicable

Availability of data and materials

The datasets used and/or analysed during the current study are available from the corresponding author on reasonable request.

Competing interests

The authors declare that they have no competing interests.

Funding

This report is independent research arising from a Doctoral Research Fellowship (DRF-2016-09-028) supported by the National Institute for Health Research (NIHR). The views expressed in this presentation are those of the authors and not necessarily those of the NHS, the National Institute for Health Research or the Department of Health and Social Care.

Authors’ contributions

MS designed the study, under supervision from TF. MS, LC and JC developed the intervention. MS collected and analysed the data. MS and TF drafted the manuscript. All authors read and approved the final manuscript.

Acknowledgements

We would like to acknowledge the valuable input from stakeholders and advisors, in particular Anne Sales for early discussions about the work and feedback about the draft animation.

References

[1] United Kingdom Research and Innovation. Research Excellence Framework. 2020. Available from: https://www.ref.ac.uk/about/

[2] National Institutes of health. Definitions of Criteria and Considerations for Research Project Grant (RPG/R01/R03/R15/R21/R34) Critiques. 2016. Available from: https://grants.nih.gov/grants/peer/critiques/rpg.htm

[3] Australian Research Council. Research Impact Principles and Framework. 2015. Available from: https://www.arc.gov.au/policies-strategies/strategy/research-impact-principles-framework

[4] Economic and Social Research Council. ESRC Social. 2021. Available from: https://www.youtube.com/channel/UCpzRsg-0wya_ao1bvb1Q9VA

[5] Canadian Institutes of Health Research. CIHR IRSC. 2021. Available from: https://www.youtube.com/c/CIHRIRSC_canada/videos

[6] Wilson PM, Petticrew M, Calnan MW and Nazareth I. Does dissemination extend beyond publication: a survey of a cross section of public funded research in the UK. Implementation Science. 2010; 5(1), pp.1-8.

[7] Research Fish. A selection of case studies from world-leading funders, charities, research organisations and research centres. 2021. Available from: https://researchfish.com/success-stories/

[8] Buykx P, Humphreys J, Wakerman J, Perkins D, Lyle D, McGrail M, Kinsman L. 'Making evidence count': A framework to monitor the impact of health services research. Australian journal of rural health. 2012 Apr;20(2):51-8.

[9] Buckarma EH, Thiels CA, Gas BL, Cabrera D, Bingener-Casey J, Farley DR. Influence of social media on the dissemination of a traditional surgical research article. Journal of surgical education. 2017 Jan 1;74(1):79-83.

[10] Maggio LA, Leroux TC, Meyer HS and Artino Jr AR. #MedEd: exploring the relationship between altmetrics and traditional measures of dissemination in health professions education. Perspectives on medical education. 2018; 7(4), pp.239-247.

[11] Nilsen P. Making sense of implementation theories, models and frameworks. Implement Sci. 2015;10:53.

[12] Baker EA, Brewer SK, Owens JS, Cook CR, Lyon AR. Dissemination Science in School Mental Health: A Framework for Future Research. School Mental Health. 2021 Apr 18:1-7.

[13] Skivington K, Craig P, Moore L, Matthews L, Simpson S. Introduction to the complex intervention concept and the research perspectives. European Journal of Public Health. 2021 Oct;31(Supplement_3):ckab164-803.

[14] O'Cathain A, Croot L, Duncan E, Rousseau N, Sworn K, Turner KM, Yardley L, Hoddinott P. Guidance on how to develop complex interventions to improve health and healthcare. BMJ open. 2019 Aug 1;9(8):e029954.

[15] McGuire WJ. Input and output variables currently promising for constructing persuasive communications. Public communication campaigns. Edited by: Rice R, Atkin C. 2001. Thousand Oaks, CA: Sage, 22-48. 3

[16] Allen HG, Stanton TR, Di Pietro F, Moseley GL. Social media release increases dissemination of original articles in the clinical pain sciences. PLoS One. 2013; 8(7):e68914.

[17] Eysenbach G. Can tweets predict citations? Metrics of social impact based on Twitter and correlation with traditional metrics of scientific impact. J Med Internet Res. 2011. Dec 19; 13(4):e123.

[18] Ashcraft LE, Quinn DA, & Brownson RC. Strategies for effective dissemination of research to United States policymakers: A systematic review. Implementation Sci. 2020; 15(1), 1–17.

[19] Johannsson H, and Selak T. Dissemination of medical publications on social media–is it the new standard? Anaesthesia. 2020; 75, 155–157.

[20] Bardus M, El Rassi R, Chahrour M, Akl EW, Raslan AS, Meho LI, Akl EA. The use of social media to increase the impact of health research: systematic review. Journal of medical Internet research. 2020;22(7):e15607.

[21] Hitlin P. Health issues topped the list of scientific studies reaching wide audiences in 2016. 2016. Available from: http://www.pewresearch.org/fact-tank/2016/12/28/health-issues-topped-the-list-of-scientific-studies-reaching-wide-audiences-in-2016/ .

[22] Tonia T, Van Oyen H, Berger A, Schindler C and Künzli N. If I tweet will you cite? The effect of social media exposure of articles on downloads and citations. International journal of public health. 2016; 61(4), pp.513-520.

[23] Fox CS, Bonaca MA, Ryan JJ, Massaro JM, Barry K, Loscalzo J. A randomized trial of social media from Circulation. Circulation. 2015 Jan 6;131(1):28-33.

[24] Thoma B, Murray H, Huang SY, Milne WK, Martin LJ, Bond CM, Mohindra R, Chin A, Yeh CH, Sanderson WB, Chan TM. The impact of social media promotion with infographics and podcasts on research dissemination and readership. Canadian Journal of Emergency Medicine. 2018 Mar;20(2):300-6.

[25] Sykes M, Thomson R, Kolehmainen N, Allan L, Finch T. Impetus to change: a multi-site qualitative exploration of the national audit of dementia. Implement Sci. 2020; Dec 15(1):1–3.

[26] Sykes M, Thomson R, Kolehmainen N, Allan L, Finch T. Opportunities to enhance ward audit: a multi-site qualitative study. BMC health services research. 2021 Dec;21(1):1-2.

[27] Research excellence framework. Index of revisions to the ‘Guidance on submissions’. 2020. Available from: https://www.ref.ac.uk/media/1447/ref-2019_01-guidance-on-submissions.pdf

[28] Wang Q, Coemans S, Siegesmund R, Hannes K. Arts-based methods in socially engaged research practice: A classification framework. Art/Research International: A Transdisciplinary Journal. 2017 Aug 23;2(2):5-39.

[29] Czajkowski SM, Powell LH, Adler N, Naar-King S, Reynolds KD, Hunter CM, Laraia B, Olster DH, Perna FM, Peterson JC, Epel E. From ideas to efficacy: The ORBIT model for developing behavioral treatments for chronic diseases. Health Psychology. 2015 Oct;34(10):971.

[30] Public Health Scotland. Statistical Process Control. 2021. Available from: https://www.isdscotland.org/Health-Topics/Quality-Indicators/Statistical-Process-Control/

[31] Oakland J and Oakland R. Statistical process control. 2018. Routledge.

[32] Scopus. Implementation Science. 2021. Available from: https://www.scopus.com/sourceid/5700191210?origin=sbrowse

[33] Implementation Science. Annual journal metrics. 2021. Available from: https://implementationscience.biomedcentral.com/

[34] BMC Health Services Research. Annual journal metrics. 2021. Available from: https://bmchealthservres.biomedcentral.com/

[35] NHS England and NHS Improvement. Making data count. 2019. Available from: https://www.england.nhs.uk/wp-content/uploads/2019/12/making-data-count-getting-started-2019.pdf

[36] McCormack L, Sheridan S, Lewis M, Boudewyns V, Melvin CL, Kistler C, Lux LJ, Cullen K and Lohr KN. Communication and dissemination strategies to facilitate the use of health-related evidence. In Database of abstracts of reviews of effects (DARE): Quality-assessed reviews. Centre for Reviews and Dissemination. 2013.

Appendix A: The brief developed by MS, LC and JC

Enhancing audit and feedback

Stylistically, the animation is to be engaging, accessible and presented with a positive, improvement-focussed tone.

Common content in both animations:

Some patients do not receive ‘best care’. To improve care, hospitals undertake ‘audit and feedback’.

Audit and feedback involves giving people a summary of their performance over a specified period of time. It is used a lot in healthcare to improve the quality of care.

Audit and feedback has different components, like assessing practice, providing feedback, making changes. These components can be done in different ways.

How these components are done can affect whether, and by how much, audit and feedback improves care.

To find ways to increase improvement from audit and feedback, we looked at how hospitals are currently doing audit and feedback. We studied six hospitals within four diverse NHS organisations We used interviews, observations and documentary analysis, and repeatedly presented emergent findings to stakeholders.

National audit:

The National Audit of dementia seeks to improve the care for people with dementia.

Almost all (98%) of hospitals take part in the National Audit of Dementia.

More than one in four people in acute hospitals have dementia but they don't always get the needed care.

We explored how the national audit of dementia is undertaken to find out how to enhance its impact on the improvement of patient care.

We studied how six hospitals undertake the national audit.

We used interviews, observations and documentary analysis, and repeatedly presented emergent findings to stakeholders.

We found that the national report was sent back to the clinical leads who then turn the national report into an internal report which it was given to Trust Level committees. The internal report described the audit and included a draft action plan.

<u>Issues</u>

We found that the feedback was slow to arrive and did not reach people providing care, instead the feedback stayed at an organisational level.

Hospital dementia leads described the national report as being confusing. They also said they were unsure how to lead hospital-wide improvement.

Hospital dementia leads used the audit report to produce an action plan for the organisation.
[Setting: Office]

The action plans focused on the care in national recommendations (e.g. improve nutritional care), often disregarding low local performance in other aspects of care (e.g. assessing risk of pressure sores).

We found that there was little exploration of why performance was low.

The clinical leads suggested actions based on what they could do (like training or more audit), rather than actions that addressed the causes of performance.

We think the hospital dementia leads need support and time to implement changes from the national audit report.

We have developed a way to support the clinical leads to improve care. We called this, 'Logical Improvement Planning'.

Logical Improvement Planning aims to help the clinical leads analyse hospital performance and consider the impact on hospital priorities.

This involves helping them to select actions based upon an analysis of the hospital data and the causes of performance. [Setting: Ward] It also involves describing what we found to influence organisational commitment to implement the changes. [Setting: Board room]

CALL TO ACTION: For more information about Logical Improvement Planning, see links on Twitter @StudyImprove or contact michael.sykes@ncl.ac.uk

Ward audit:

Ward audit is a form of audit and feedback which involves ward managers regularly receiving feedback about the clinical performance of staff on their ward.

Ward audit feedback includes information about the performance of clinicians at things like:

- medicine management (e.g. recording the giving of drugs),
- infection control (e.g. checking if the ward has been cleaned),
- nutrition (e.g. whether patient needs have been assessed),
- bladder and bowel care (e.g. writing when a patient's catheter was inserted)
- communication (e.g. whether discharge has been discussed with carers or relatives). [Setting: Patient bedside]

We explored how ward audit is undertaken to find out how to enhance its impact on the improvement of patient care.

We found that at three sites clinical staff extracted data from the clinical record, and sometimes did interviews with patients or staff and observed how care was given. Ward managers knew the results when they were entered, and got analysed data within a week. The analysed data was colour coded, typically red, amber and green.

<u>Issues</u>

At one of the hospitals, it was noticed that manually gathering the data took 5 hours per ward per month. This was done by band 5 to 7 nurses. Staff prioritised data collection rather than taking care of the patients. [Setting: On ward e.g. Looking in patient notes / speaking / observing staff and patients]

The data that was collected was not always accurate, a reason for this was the fear of feedback.

The feedback that got back to the staff was often received as punitive. [Setting: Office]

- "it would be like the house of cards that was toppled from the top and it would be all down. So, [matron] would be under pressure and she would put that pressure on me" (Ward manager)
- "We were told it…was a massive failure on our part as a ward." (staff nurse)
- Staff at different levels did not think that the data was accurate. This meant that the data was not used to develop improvement actions.
- To improve the data hospitals:
- Audited the same topic in a different way, for example, by the infection control nurse.
- Bought electronic tablets to record the data more quickly
- Introduced 'ward accreditation', where a member of staff who was not from the ward collected data. [Setting: Office]

A requirement to undertake 'low value' work may undermine staff attitudes towards the quality of care. Punitive feedback may decrease staff morale, increase staff turnover and reduce the quality of care. [Setting: Ward]

<u>Solutions</u>

We propose an intervention to:

- discuss the accuracy of ward monitoring data and the extent it leads to changes likely to improve care,
- explore local perceptions of the feedback within ward monitoring audit and other forms of clinical performance management,
- explore causes of punitive feedback, [Setting: Office]
- consider implications from punitive feedback (e.g. staff morale, collective leadership, ability to find high-performing wards that might have lessons to share, ability to develop hospital-level interventions) [Setting: Committee table]

CALL TO ACTION: There is an opportunity to improve care by changing the way ward monitoring feedback is given. To get involved in a study to enhance ward audit, see links on Twitter @StudyImprove or contact michael.sykes@ncl.ac.uk

AT THE END OF EACH:

Research funded by the National Institute for Health Research (NIHR) (Michael Sykes' Doctoral Fellowship 2016-09-028)

This publication presents independent research funded by the National Institute for Health Research (NIHR). The views expressed are those of the author and not necessarily those of the NHS, the NIHR or the Department of Health and Social Care.

[LOGO ATTACHED]

Appendix 2:

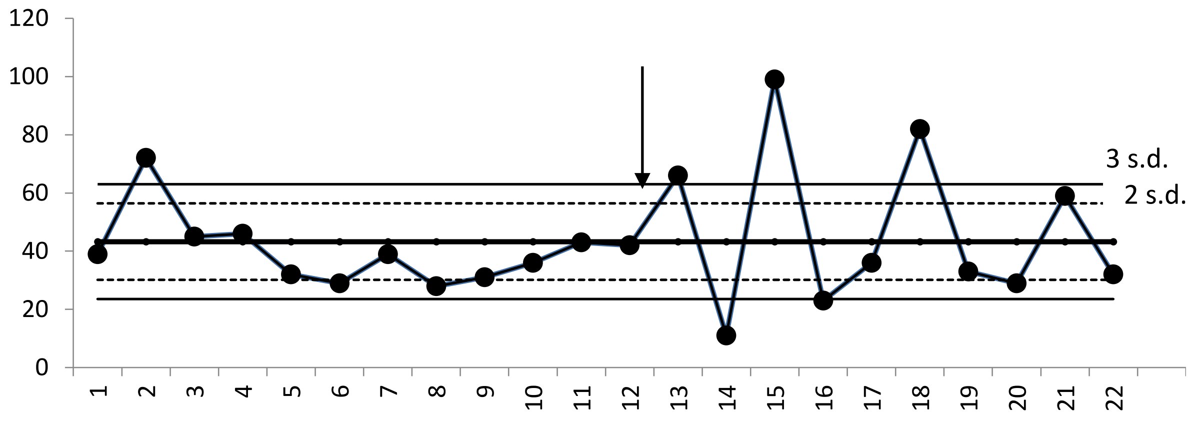

Figure 3: The unadjusted statistical process control c-chart showing weekly accesses of Sykes et al (2021)

Appendix B: CONSORT 2010 checklist of information to include when reporting a randomised trial*

Section/Topic	Item No	Checklist item	Reported on page No
**Title and abstract**			
	1a	Identification as a randomised trial in the title	N/A
	1b	Structured summary of trial design, methods, results, and conclusions (for specific guidance see CONSORT for abstracts)	1
**Introduction**			
Background and objectives	2a	Scientific background and explanation of rationale	1
	2b	Specific objectives or hypotheses	2
**Methods**			
Trial design	3a	Description of trial design (such as parallel, factorial) including allocation ratio	2
	3b	Important changes to methods after trial commencement (such as eligibility criteria), with reasons	3
Participants	4a	Eligibility criteria for participants	3
	4b	Settings and locations where the data were collected	3
Interventions	5	The interventions for each group with sufficient details to allow replication, including how and when they were actually administered	3
Outcomes	6a	Completely defined pre-specified primary and secondary outcome measures, including how and when they were assessed	4
	6b	Any changes to trial outcomes after the trial commenced, with reasons	N/A
Sample size	7a	How sample size was determined	N/A
	7b	When applicable, explanation of any interim analyses and stopping guidelines	N/A
Randomisation:			
Sequence generation	8a	Method used to generate the random allocation sequence	N/A
	8b	Type of randomisation; details of any restriction (such as blocking and block size)	N/A
Allocation concealment mechanism	9	Mechanism used to implement the random allocation sequence (such as sequentially numbered containers), describing any steps taken to conceal the sequence until interventions were assigned	N/A

Implementation	10	Who generated the random allocation sequence, who enrolled participants, and who assigned participants to interventions	N/A
Blinding	11a	If done, who was blinded after assignment to interventions (for example, participants, care providers, those assessing outcomes) and how	N/A
	11b	If relevant, description of the similarity of interventions	4
Statistical methods	12a	Statistical methods used to compare groups for primary and secondary outcomes	4
	12b	Methods for additional analyses, such as subgroup analyses and adjusted analyses	
**Results**			
Participant flow (a diagram is strongly recommended)	13a	For each group, the numbers of participants who were randomly assigned, received intended treatment, and were analysed for the primary outcome	5
	13b	For each group, losses and exclusions after randomisation, together with reasons	5
Recruitment	14a	Dates defining the periods of recruitment and follow-up	5-6
	14b	Why the trial ended or was stopped	N/A
Baseline data	15	A table showing baseline demographic and clinical characteristics for each group	N/A
Numbers analysed	16	For each group, number of participants (denominator) included in each analysis and whether the analysis was by original assigned groups	N/A
Outcomes and estimation	17a	For each primary and secondary outcome, results for each group, and the estimated effect size and its precision (such as 95% confidence interval)	4
	17b	For binary outcomes, presentation of both absolute and relative effect sizes is recommended	N/A
Ancillary analyses	18	Results of any other analyses performed, including subgroup analyses and adjusted analyses, distinguishing pre-specified from exploratory	5
Harms	19	All important harms or unintended effects in each group (for specific guidance see CONSORT for harms)	N/A
**Discussion**			
Limitations	20	Trial limitations, addressing sources of potential bias, imprecision, and, if relevant, multiplicity of analyses	6
Generalisability	21	Generalisability (external validity, applicability) of the trial findings	6
Interpretation	22	Interpretation consistent with results, balancing benefits and harms, and considering other relevant evidence	6
**Other information**			
Registration	23	Registration number and name of trial registry	4

Protocol	24	Where the full trial protocol can be accessed, if available	N/A
Funding	25	Sources of funding and other support (such as supply of drugs), role of funders	8 [Note: No incentives were provided to participants]

*We strongly recommend reading this statement in conjunction with the CONSORT 2010 Explanation and Elaboration for important clarifications on all the items. If relevant, we also recommend reading CONSORT extensions for cluster randomised trials, non-inferiority and equivalence trials, non-pharmacological treatments, herbal interventions, and pragmatic trials. Additional extensions are forthcoming: for those and for up to date references relevant to this checklist, see www.consort-statement.org.

Appendix C: The TIDieR (Template for Intervention Description and Replication) Checklist*:

Item number	Item	Where located ** Primary paper (page or appendix number)	Other † (details)
	**BRIEF NAME**		
**1.**	Provide the name or a phrase that describes the intervention.	______1______	______________
	**WHY**		
**2.**	Describe any rationale, theory, or goal of the elements essential to the intervention.	______2______	______________
	**WHAT**		
**3.**	Materials: Describe any physical or informational materials used in the intervention, including those provided to participants or used in intervention delivery or in training of intervention providers. Provide information on where the materials can be accessed (e.g. online appendix, URL).	______3______	______________
**4.**	Procedures: Describe each of the procedures, activities, and/or processes used in the intervention, including any enabling or support activities.	______4______	______________
	**WHO PROVIDED**		
**5.**	For each category of intervention provider (e.g. psychologist, nursing assistant), describe their expertise, background and any specific training given.	______3______	______________
	**HOW**		
**6.**	Describe the modes of delivery (e.g. face-to-face or by some other mechanism, such as internet or telephone) of the intervention and whether it was provided individually or in a group.	______3______	______________
	**WHERE**		

**7.**	Describe the type(s) of location(s) where the intervention occurred, including any necessary infrastructure or relevant features.	______3_______	_____________
	**WHEN and HOW MUCH**		
**8.**	Describe the number of times the intervention was delivered and over what period of time including the number of sessions, their schedule, and their duration, intensity or dose.	______4______	_____________
	**TAILORING**		
**9.**	If the intervention was planned to be personalised, titrated or adapted, then describe what, why, when, and how.	______N/A____	_____________
	**MODIFICATIONS**		
**10.**‡	If the intervention was modified during the course of the study, describe the changes (what, why, when, and how).	_____N/A_____	_____________
	**HOW WELL**		
**11.**	Planned: If intervention adherence or fidelity was assessed, describe how and by whom, and if any strategies were used to maintain or improve fidelity, describe them.	_____N/A____	_____________
**12.**‡	Actual: If intervention adherence or fidelity was assessed, describe the extent to which the intervention was delivered as planned.	______N/A____	_____________

** **Authors** - use N/A if an item is not applicable for the intervention being described. **Reviewers** – use '?' if information about the element is not reported/not sufficiently reported.

† If the information is not provided in the primary paper, give details of where this information is available. This may include locations such as a published protocol or other published papers (provide citation details) or a website (provide the URL).

‡ If completing the TIDieR checklist for a protocol, these items are not relevant to the protocol and cannot be described until the study is complete.

Figure 1

Click here to access/download;Figure;Animation Figure 1 .docx

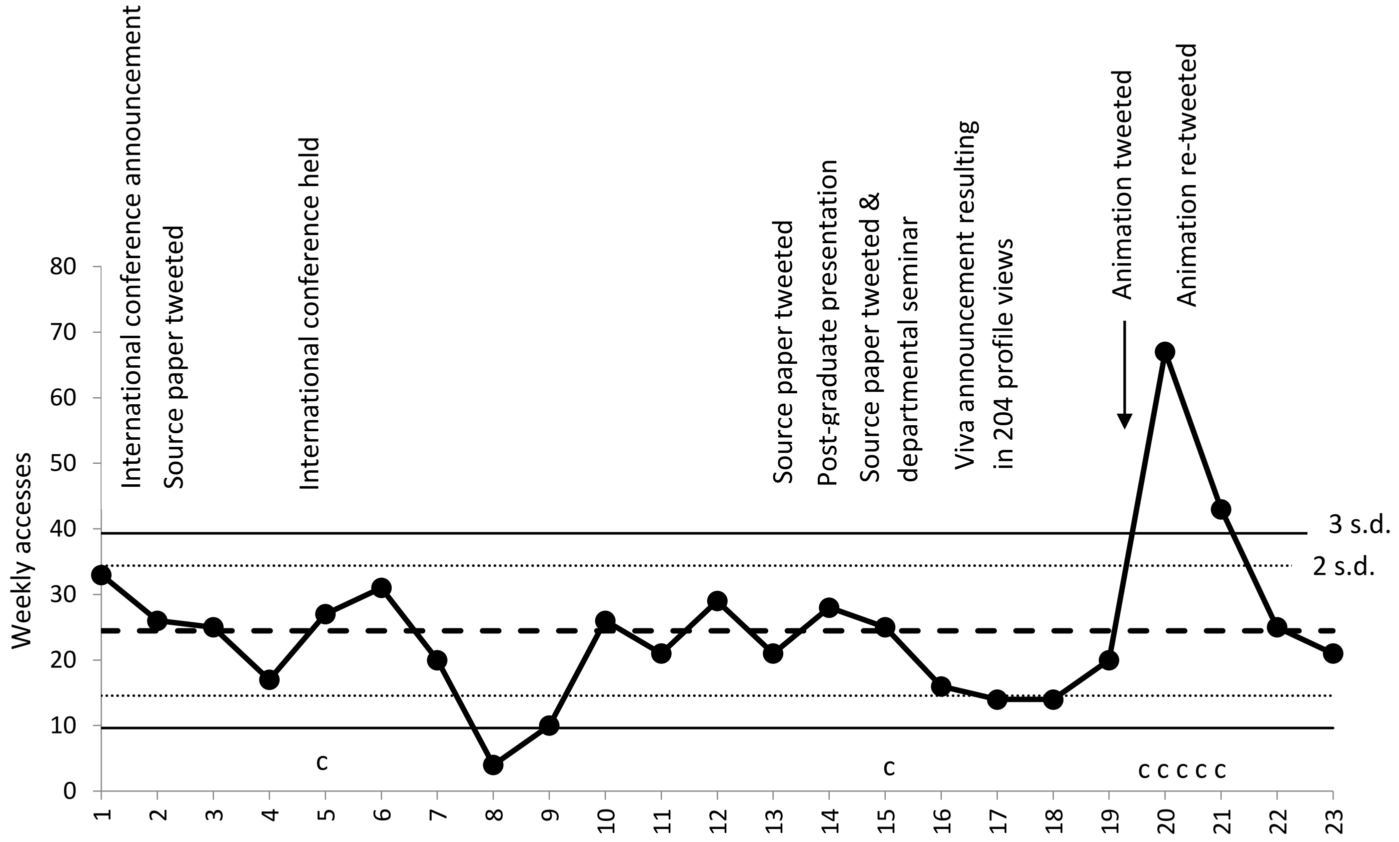

Figure 1

Click here to access/download;Figure;Animation Figure 2.docx

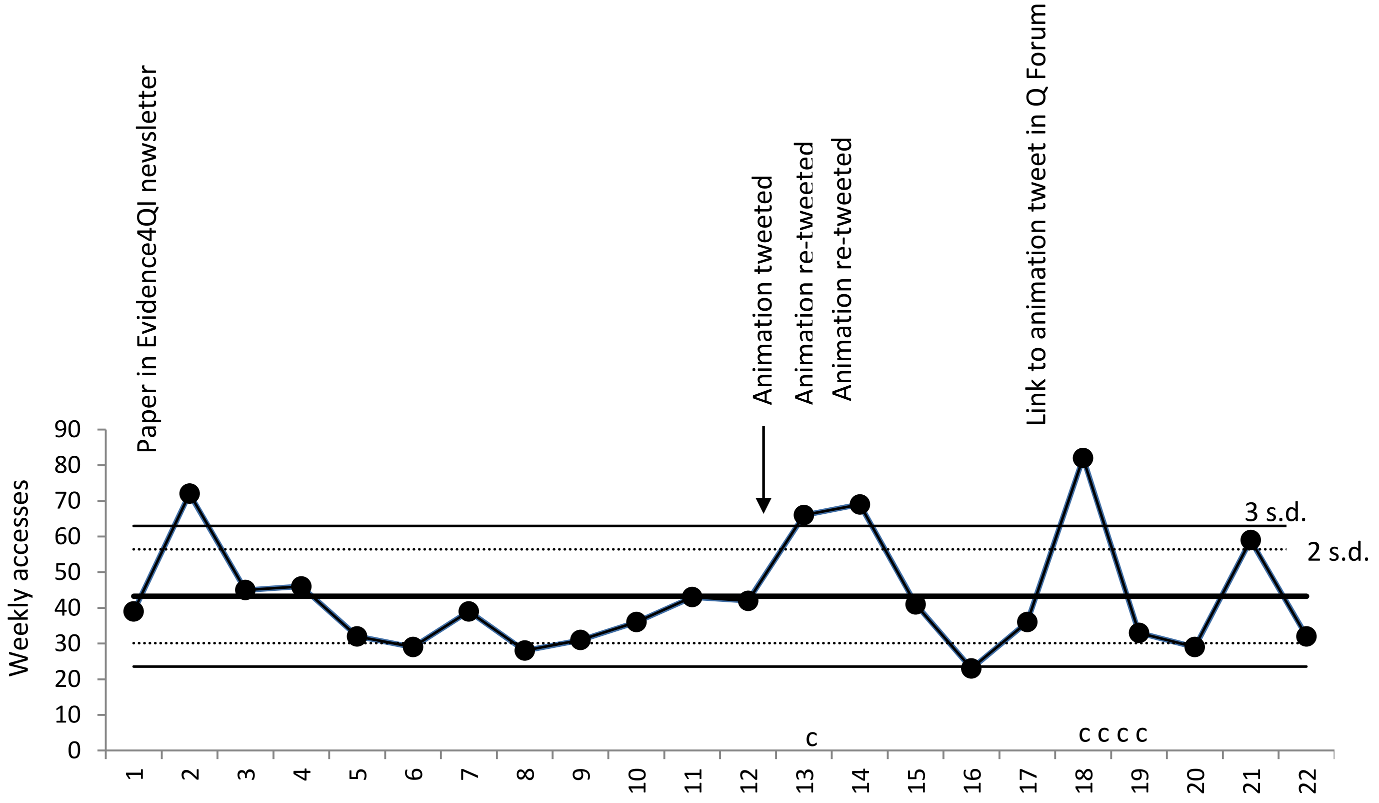

Figure 2
